# Supplementary material for: Bacterial colonization stimulates a complex physiological response in the immature human intestinal epithelium
Source: eLife. 2017 Nov 7;6:e29132. doi: 10.7554/eLife.29132 (PMC5711377; doi:10.7554/eLife.29132)
Supplement: Transparent reporting form [file elife-29132-transrepform.docx]

***eLife’s* transparent reporting form**

We encourage authors to provide detailed information *within their submission* to facilitate the interpretation and replication of experiments. If you have any questions, please contact us: [editorial@elifesciences.org](mailto:editorial@elifesciences.org).

**Sample-size estimation**

- You should state whether an appropriate sample size was computed when the study was being designed
- You should state the statistical method of sample size computation and any required assumptions
- If no explicit power analysis was used, you should describe how you decided what sample (replicate) size (number) to use

Please outline where this information can be found within the submission (e.g., page numbers or figure legends), or explain why this information doesn’t apply to your submission:

Sample sizes are indicated in the figure legends where applicable. In the majority of cases, sample size was determined by the practical limitations of the culture system and measurement techniques. Where representative imaging data is shown, the images selected where chosen to represent at least 3 independent experiments. RNA-seq experiments include 3-6 experimental replicates per treatment condition. A complete record of the RNA-seq dataset can be found at the ArrayExpress database E-MTAB-5801.

**Replicates**

- You should report how often each experiment was performed
- You should include a definition of biological versus technical replication
- The data obtained should be provided and sufficient information should be provided to indicate the number of independent biological and/or technical replicates
- If you encountered any outliers, you should describe how these were handled
- Criteria for exclusion/inclusion of data should be clearly stated
- High-throughput sequence data should be uploaded before submission, with a private link for reviewers provided (these are available from both GEO and ArrayExpress)

Please outline where this information can be found within the submission (e.g., page numbers or figure legends), or explain why this information doesn’t apply to your submission:

Source data and all analysis code will be made publicly available at https://github.com/hilldr/Hill_HIO_Colonization_2017, as indicated on page 21. This includes complete documentation of experimental replicates and data processing. As a rule, outliers were not excluded from any part of the analysis. A complete record of the RNA-seq dataset can be found at the ArrayExpress database E-MTAB-5801.

**Statistical reporting**

- Statistical analysis methods should be described and justified
- Raw data should be presented in figures whenever informative to do so (typically when N per group is less than 10)
- For each experiment, you should identify the statistical tests used, exact values of N, definitions of center, methods of multiple test correction, and dispersion and precision measures (e.g., mean, median, SD, SEM, confidence intervals; and, for the major substantive results, a measure of effect size (e.g., Pearson's r, Cohen's d)
- Report exact p-values wherever possible alongside the summary statistics and 95% confidence intervals. These should be reported for all key questions and not only when the p-value is less than 0.05.

The methods section includes a description of the statistical analyses on page 21. Throughout the paper, P- values are reported exactly and statistical tests are described in figure legends where appropriate. Source data and all analysis code will be made publicly available at https://github.com/hilldr/Hill_HIO_Colonization_2017. The analysis code contains explicit documentation of all statistical tests applied in this study, the results of which can be reproduced directly from the raw data available at the same address.

Please outline where this information can be found within the submission (e.g., page numbers or figure legends), or explain why this information doesn’t apply to your submission:

(For large datasets, or papers with a very large number of statistical tests, you may upload a single table file with tests, Ns, etc., with reference to page numbers in the manuscript.)

**Additional data files (“source data”)**

- We encourage you to upload relevant additional data files, such as numerical data that are represented as a graph in a figure, or as a summary table
- Where provided, these should be in the most useful format, and they can be uploaded as “Source data” files linked to a main figure or table
- Include model definition files including the full list of parameters used
- Include code used for data analysis (e.g., R, MatLab)
- Avoid stating that data files are “available upon request”

Please indicate the figures or tables for which source data files have been provided:

Source data and all analysis code will be made publicly available at https://github.com/hilldr/Hill_HIO_Colonization_2017. The analysis code is designed to reproduce the entire manuscript from the raw data files. This information is also available on page 21 of the manuscript.
